# A Robust Deep-Learning Model for Landslide Susceptibility Mapping: A Case Study of Kurdistan Province, Iran

**DOI:** 10.3390/s22041573

**Published:** 2022-02-17

**Authors:** Bahareh Ghasemian, Himan Shahabi, Ataollah Shirzadi, Nadhir Al-Ansari, Abolfazl Jaafari, Victoria R. Kress, Marten Geertsema, Somayeh Renoud, Anuar Ahmad

**Affiliations:** 1 Department of Geomorphology, Faculty of Natural Resources, University of Kurdistan, Sanandaj 6617715175, Iran; b.ghassemian@nr.uok.ac.ir; 2 Department of Rangeland and Watershed Management, Faculty of Natural Resources, University of Kurdistan, Sanandaj 6617715175, Iran; a.shirzadi@uok.ac.ir; 3 Civil, Environmental and Natural Resources Engineering, Lulea University of Technology, 97187 Lulea, Sweden; nadhir.alansari@ltu.se; 4 Research Institute of Forests and Rangelands, Agricultural Research, Education and Extension Organization (AREEO), Tehran 1496813111, Iran; jaafari@rifr-ac.ir; 5 Department of Ecosystem Science and Management, University of Northern British Columbia, 3333 University Way, Prince George, BC V2N 4Z9, Canada; kressv@unbc.ca; 6 Research Geomorphologist, Ministry of Forests, Lands, Natural Resource Operations and Rural Development, 499 George Street, Prince George, BC V2L 1R5, Canada; Marten.Geertsema@gov.bc.ca; 7 Data Mining Laboratory, Department of Engineering, College of Farabi, University of Tehran, Tehran 1417935840, Iran; somayehronoud@gmail.com; 8 Department of Geoinformation, Faculty of Built Environment and Surveying, Universiti Teknologi Malaysia (UTM), Johor Bahru 81310, Malaysia; anuarahmad@utm.my

**Keywords:** landslide susceptibility, extreme learning machine, deep belief network, genetic algorithm, GIS, Iran

## Abstract

We mapped landslide susceptibility in Kamyaran city of Kurdistan Province, Iran, using a robust deep-learning (DP) model based on a combination of extreme learning machine (ELM), deep belief network (DBN), back propagation (BP), and genetic algorithm (GA). A total of 118 landslide locations were recorded and divided in the training and testing datasets. We selected 25 conditioning factors, and of these, we specified the most important ones by an information gain ratio (IGR) technique. We assessed the performance of the DP model using statistical measures including sensitivity, specificity, accuracy, F1-measure, and area under-the-receiver operating characteristic curve (AUC). Three benchmark algorithms, i.e., support vector machine (SVM), REPTree, and NBTree, were used to check the applicability of the proposed model. The results by IGR concluded that of the 25 conditioning factors, only 16 factors were important for our modeling procedure, and of these, distance to road, road density, lithology and land use were the four most significant factors. Results based on the testing dataset revealed that the DP model had the highest accuracy (0.926) of the compared algorithms, followed by NBTree (0.917), REPTree (0.903), and SVM (0.894). The landslide susceptibility maps prepared from the DP model with AUC = 0.870 performed the best. We consider the DP model a suitable tool for landslide susceptibility mapping.

## 1. Introduction

Landslides occur in a variety of materials and undergo various styles of movement at different rates [1]. Landslides play an important geomorphological role in the evolution of landscapes, impacting the natural (soils, ecosystems, aquatic habitat, etc.) and built (residential areas, roads, pipelines, etc.) environment [2,3]. Landslide hazards are often exacerbated by land use practices such as road building, and deforestation, and may be made worse by increases in precipitation [4]. Therefore, it is important to identify areas that have a high potential for landslides and mitigate landslide damage.

Landslide risk assessment methodologies can be classified into three dominant groups: qualitative, quantitative, and artificial intelligence approaches. Qualitative approaches often rely on air photo and field interpretation and expert judgment (e.g., Schwab and Geertsema [5]). Quantitative methods are based on mathematical rules and expert judgment [6]. Artificial intelligence techniques can use subjective knowledge or pattern recognition techniques to solve a set of mathematical equations. Selection of the most appropriate model is usually based on the type of data available, the scale of the case study and analysis, and the knowledge of the researcher [7].

In recent decades, with the rapid development of geographic information systems (GIS) remote sensing (RS) techniques and improvements in the computing power of artificial intelligence algorithms, machine learning has played an important role in increasing the accuracy and reliability of landslide predictions [8]. Machine learning methods depend on field observations and statistical calculations [9]. Machine learning uses computer algorithms for analyzing and forecasting information by learning training datasets [10]. They have a high ability to detect landslide occurrence behavior using distribution estimation algorithms, they have a data-driven nature, and they utilize high repetition of the modeling process. In several studies, these methods have proven their comparative advantage over bivariate and multivariate statistical models [11,12].

Several machine-learning methods have been applied in landslide susceptibility assessment, such as logistic regression (LR) [13], naive Bayes (NB) [14,15], fuzzy logic (FL) [16], support vector machines (SVM) [17,18,19], kernel logistic regression (KLR) [20,21], Bayesian logistic regression (BLR) [17,22], artificial neural network (ANN) [23,24], random forest (RF) [25,26,27,28], rotation forest [29,30], random subspace (RS) [31], neuro-fuzzy inference system (ANFIS) [32,33], decision tree (DT) [26], classification and regression tree (CART) [34,35,36] and many other methods [37].

Despite the logical results and high performance of different models, geoscientists are always looking for new methods to more accurately identify landslide-prone areas and produce reliable maps needed for environmental planning. Therefore, presenting a new approach based on artificial intelligence algorithms, deep learning and GIS-RS techniques for landslide modeling is of high necessity in landslide hazards management [38].

One of the major challenges in mountainous areas is the occurrence of landslides, of which the occurrence is naturally inevitable and cannot be completely prevented but can be managed. In this study, we are looking for a technique that can be combined with several methods to achieve an algorithm with higher predictive power than conventional machine-learning algorithms to predict landslide prone areas. Despite the logical results and high performance of different models, geoscientists are always looking for new methods with quantitative criteria to more accurately identify landslide-prone areas and reliable maps needed for environmental planning. Therefore, presenting a new approach based on artificial intelligence algorithms and remote sensing techniques for landslide modeling is of high necessity in landslide management. We applied and developed a deep-learning model based on ELM, DBN, and BP optimized by GA for landslide susceptibility mapping. This model has been used earlier in spatial prediction against floods [39] and also has been applied to predict cancer [40]; however, its ability has not been evaluated to landslide susceptibility mapping so far. The model has been confirmed by some statistical measures and compared with some state-of-the-art benchmark machine-learning algorithms including SVM, NBTree and REPTree. We developed this model in MATLAB 2018a and all landslide susceptibility maps were produced in ArcGIS 10.5. The purpose of this study is to evaluate a robust deep-learning model that will support landslide susceptibility mapping. Here, we build on previous landslide susceptibility modelling for this study area using landslide data belonging to Asadi et al. [41], but with a different set of algorithms.

## 2. Study Area and Data

### 2.1. Description of the Study Area

**Figure 1:**
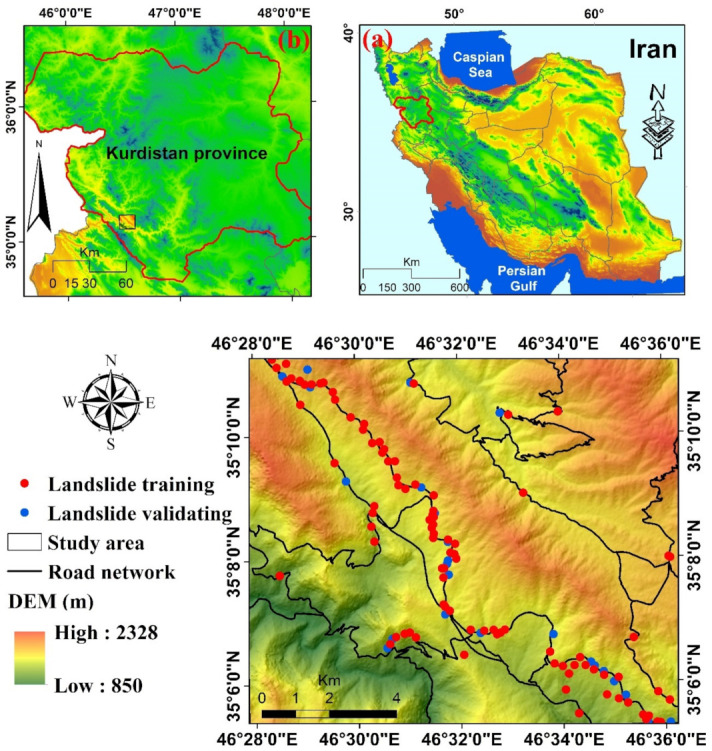
Geographical location of the study area in (**a**) Iran and (**b**) Kurdistan province.

Our study area, around Kamyaran city, is a mountainous area of nearly 150 km^2^ in the southwest of the Kurdistan Province, Iran (Figure 1). The elevation ranges from 850 to 2328 m and has a mean annual temperature that varies between 11.3 °C and 17 °C and mean annual precipitation of 528 mm. Geologically, the study area is in the Sirvan drainage basin, located in the structural zone of Sanandaj-Sirjan and Zagros. Bedrock lithologies include outcrops from Cretaceous to Quaternary rocks, the oldest of which include Micrite limestone and dark gray shale. Most of the study areas are covered by Mesozoic and Cretaceous formations, which include Basaltic pillow lava and dark grey shale with intercalations of volcanic rocks. Holocene sediments of the Old Testament include alluvial fans and alluvial barracks. The predominant land covers in the study area are semi-dense forests and dry farming. In addition, dense pasture, semi-dense pasture, low-dense forest and woodlands are other types of land cover/land use in the study area. The area is significantly prone to landslides associated with road developments.

### 2.2. Data

#### 2.2.1. Landslide Inventory Map

It is necessary to prepare a landslide distribution map for landslide modeling because the assumption of the modeling process is that future landslides occur in the same conditions as in the past [42]. That “*the past and the present are key to the future*” is one of the most important principles in earth science. This means landslides that have occurred in the past and present under specific topographic, geological, hydrogeological, and climatic conditions in an area can provide useful information to predict the potential for future landslides in that area [43]. A map showing such information is useful for studying the spatial relationships between landslide distribution and factors affecting landslide occurrence [44]. Galli et al. [45] have mentioned that the quality of a landslide inventory map can lead to reasonable results in landslide modeling. From a total of 118 landslide points detected in the study area, 94 points (~80%) were used as the training dataset, and 24 points (~20%) were considered as the validation dataset. A total of 118 landslide locations used in this study were a part of a total of 175 landslide locations of Asadi et al. [41].

#### 2.2.2. Landslide Conditioning Factors

**Figure 2:**
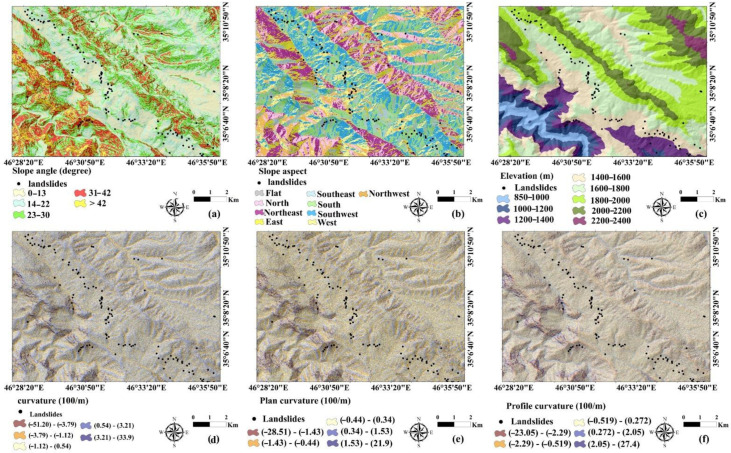

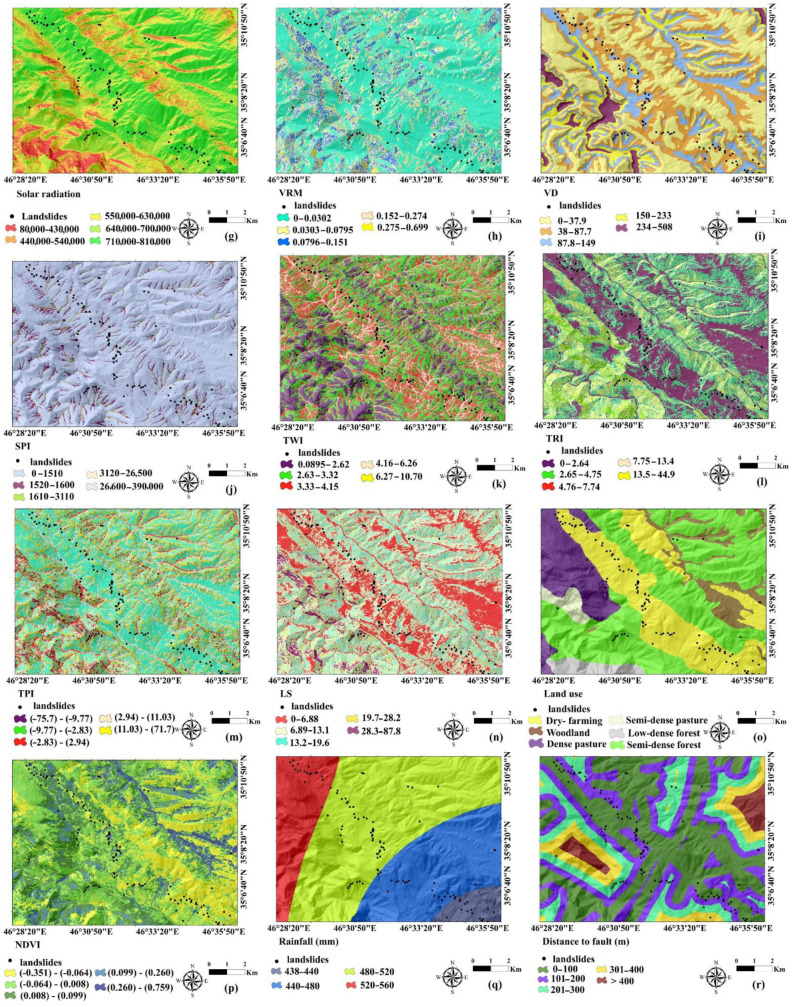

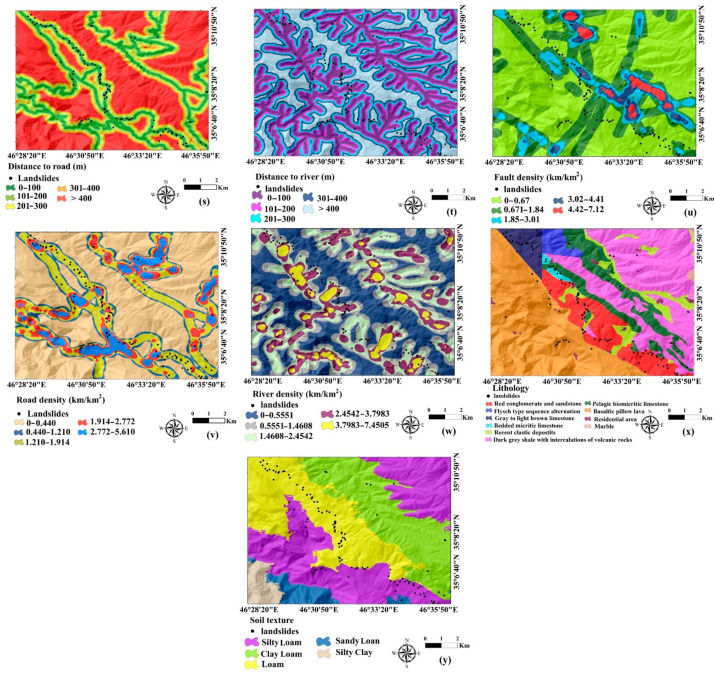
Landslide conditioning factors used in this study: (**a**) slope angle, (**b**) aspect, (**c**) elevation, (**d**) curvature, (**e**) plan curvature, (**f**) profile curvature, (**g**) solar radiation, (**h**) VRM, (**i**) VD, (**j**) SPI, (**k**) TWI, (**l**) TRI, (**m**) TPI, (**n**) LS, (**o**) land use, (**p**) NDVI, (**q**) rainfall, (**r**) distance to fault, (**s**) distance to road, (**t**) distance to river (**u**), fault density, (**v**) road density, (**w**), river density, (**x**) lithology, and (**y**) soil texture.

The selection of the factors affecting the occurrence of landslides is one of the most important steps in landslide susceptibility studies [46]. In this study, we selected 25 conditioning factors that were slope angle, aspect, elevation above sea level, curvature, profile curvature, plan curvature, solar radiation, valley depth (VD), terrain ruggedness index (TRI), vector ruggedness measure (VRM), stream power index (SPI), topographic wetness index (TWI), length slope (LS), topographic position index (TPI), land use, normalized difference vegetation index (NDVI), lithology, soil, distance to fault, distance to river, distance to road, fault density, river density, road density, and rainfall (Figure 2). We used some conditioning factors for this study area that were earlier published by Asadi et al. (2022).

##### Slope Angle

Landslide hazard is often linked to slope angle, with shear stresses increasing on steeper slopes [47]. The supply of soil available for sliding often thins dramatically on steeper slopes above 25 degrees [48]. In other words, on high slopes, the type of material is more often stone and outcrops, such that medium slopes are more prone to landslides. This layer in the present study was extracted from the digital elevation model (DEM) and classified into eight intervals: 0–13, 14–22, 23–30, 31–42, and >43 (Figure 2a).

##### Aspect

Slope direction affects the occurrence of landslides by controlling the parameters related to soil moisture concentration, sunlight, dry winds, rainfall (saturation degree), and discontinuities [49]. This layer was extracted from DEM and categorized into nine classes: flat (−1–39.08), north (39.08–79.16), northeast (79.16–119.24), east (119.246–159.32), southeast (159.32–199.41), south (199.41–239.49), southwest (239.49–279.57), west (279.57–319.65), and northwest (319.65–359.74) (Figure 2b).

##### Elevation

The influences of elevation on landslides are often displayed as indirect relationships or by means of other factors [50]. The altitude factor of each region is one of the effective layers in creating slope instabilities. This factor indirectly determines many causes of landslides such as annual rainfall, heavy rainfall, temperature, frost changes, ice melting, etc. [51]. Maximum elevation of the region is 2328 m, and the minimum elevation is 850 m, hence the general elevation variance is 1478 m. The elevation map was extracted from DEM and then classified into eight classes: (1) 850–1000, (2) 1000–1200, (3) 1200–1400, (4) 1400–1600, (5) 1600–1800, (6) 1800–2000, (7) 2000–2200, and (8) 2200–2400 (Figure 2c).

##### Curvature

Curvature maps show the extent to which the surface deviates from the flatness, or in other words, the convexity and concaveness of the slope [52]. The curvature of the slope represents the shape of the topography so that the positive concavity represents the surface where the pixels are convex (Convex, Coves, Hollows), Negative concavity indicates a surface where the pixels are concave (Concave, Noses) and zero indicates a surface that has no slope and is straight (Flat, Straight). These three types of slope shapes have a great effect on slope instability by controlling the concentration and diffusion of surface and subsurface water in the slopes [53]. Convexity and concavity of the slope curvature map using distances between consecutive topographic lines in the GIS were extracted from the DEM of the region and classified into five classes (1) highly concave (−51.20)–(−3.79), concave (−3.79)–(−1.12), (3) flat (−1.12)–(0.54), (4) convex (0.54)–(3.21) and (5) very convex (3.21)–(33.9) (Figure 2d).

##### Plan Curvature

Plan curvature indicates changes in direction along a curve. This factor affects the divergence and convergence of water and materials containing a landslide in the path of motion. Plan curvature was extracted from DEM and divided into five classes: (1) [(−28.51)–(−1.43)], (2) [(−1.43)–(−0.44)], (3) [(−0.44)–(0.34)], (4) [(0.34)–(1.53)], and (5) [(1.53)–(21.09)] (Figure 2e).

##### Profile Curvature

Profile curvature is an important factor that affects the stress resistance due to landslides in the path and indicates the intensity of water flow and transportation and deposition processes [54]. The positive values in the transverse curvature of the slope indicate concavity (decrease in flow rate) and the negative values indicate convexity (increase in flow rate) [55]. Profile curvature was extracted from DEM and constructed in five categories: (1) [(−23.05)–(−2.29)], (2) [(−2.29)–(−0.519)], (3) [(−0.519)–(0.272)], (4) [(0.272)–(2.05)], and (5) [(2.05)–(27.4)] (Figure 2f).

##### Solar Radiation

The average convergence of solar radiation per pixel over a year is called the intensity of solar radiation, which is expressed in kilowatt hours per square meter [56]. The importance of this index is that its larger value indicates more vapor than the soil surface in an area. This index also controls the amount of vegetation on the slope. The less solar radiation that reaches a slope, the more vegetation appears on the slope, and as a result, the slope becomes more stable [57,58]. In the present study, the solar radiation layer was extracted from DEM in ArcGIS and categorized into five classes: (1) 80,000–43,000, (2) 440,000–540,000, (3) 550,000–630,000, (4) 640,000–700,000, and (5) 710,000–810,000 (Figure 2g).

##### Vector Ruggedness Measure

Vector ruggedness measure (VRM) factor was suggested by Hobson et al. [59]. It provides a way to measure terrain ruggedness as the variation in the three-dimensional orientation of grid cells within a neighborhood: slope and aspect are captured into a single measure and used to decouple terrain ruggedness from just slope or elevation [60]. The VRM map was created from DEM in the SAGA GIS software environment and then it was divided into five classes: (1) 0–0.0302, (2) 0.0303–0.0795, (3) 0.796–0.151, (4) 0.152–0.274, and 0.275–0.699 (Figure 2h).

##### Valley Depth

The valley depth (VD) factor can also be considered one of the fundamental layers in assessing landslide susceptibility. This index was prepared based on DEM map in the SAGA GIS software, and after exporting to ArcGIS it was classified into five classes: (1) 0–37.9, (2) 38–87.7, (3) 87.8–149, and (4) 150–233 and (5) 234–508 m (Figure 2i).

##### Stream Power Index

Stream Power Index (SPI) is a criterion derived from the DEM that might affect landslide occurrence, and it reflects the erosive power of slope surface run-off [61,62]. It can be formulated as follows:

(1) $$\mathrm{SPI}={\;A}_{s}\times \tan\beta \;$$

where $A_{s}$ is the specific basin area and tanβ represents the slope angle. In this study, it was prepared based on DEM in the SAGA GIS software and then exported to ArcGIS software to map. The SPI layer was then extracted in five intervals: (1) 0–1510, (2) 1520–1600, (3) 1610–3110, (4) 3120–26,500, (5) 26,600–390,000 (Figure 2j).

##### Topographic Wetness Index

Topographic wetness index (TWI) represents a theoretical component of flow accumulation at any point in a watershed or region that is used to describe the spatial pattern of soil moisture [63]. This index is generally used for topographic control over hydrological processes and its high values are generally used in landslide bodies. The TWI can be formulated as follows:

(2) $$\mathrm{TWI}=\mathrm{Ln}\left(\frac{A_{s}}{\tan\beta }\right)$$

where $A_{s}$ is cumulative drainage upstream area at one point and tan the angle of slope at the point. The TWI was prepared in five classes: (1) 0.0895–2.62, (2) 2.63–3.32, (3) 3.33–4.15, (4) 4.16–6.26, and (5) 6.26–10.70 (Figure 2k).

##### Terrain Ruggedness Index

Terrain Ruggedness Index (TRI) was introduced by Riley [64], and it is actually the difference in the height of one pixel with the eight pixels around it. Equation (3) is provided to calculate this index:

(3) $$\mathrm{TRI}=\surd \overset{8}{\underset{p=1}{\sum }}ZMD$$

where *p* is the number of pixels in the region and *ZMD* is the average difference of eight pixels around each pixel. The TRI map was prepared in five classes: (1) 0–2.64, (2) 2.65–4.75, (3) 4.76–7.74, (4) 7.75–13.4, and 13.5–44.9 (Figure 2l).

##### Topographic Position Index

Topographic position index (TPI) compares the height of each pixel in the digital elevation model with the specified pixel around that pixel [65]. To calculate TPI (Equation (4)), the height of each cell in a digital elevation model compared with the average height of neighboring cells is examined. Finally, the average height decreases from the height value in the center. Areas higher than the surrounding points (hills) are indicated by positive TPI values; negative TPI values denote areas lower than their surroundings (valleys). Zero and near-zero values also illustrate flat areas (where the slope is close to zero) or areas with a fixed slope [66].

(4) TPI= Z0−∑n−1Znn

where $Z_{0}$ is the point height of the model under evaluation, $Z_{n}$ is the height of the grid and *n* is the total number of surrounding points considered in the evaluation. We prepared TPI in five classes: (1) (−75.7)–(−9.77), (2) (−9.77)–(−2.83), (3) (−2.83)–(2.94), (4) (2.94)–(11.03), and (5) (11.03)–(71.7) (Figure 2m).

##### Slope Length

The slope length (LS) factor, which is a combination of the slope angle and length of the slope, is a fundamental factor in the study of landslides because this factor refers to the sediment transport capacity created by the landslide through the daily (direct) flow. Carrara [67] stated that there is a relationship between landslide density and slope length. Therefore, this factor is examined in this study [67]. Mathematically, this equation is expressed as:

(5) $$\mathrm{LS}={\left(\frac{A_{s}}{22.13}\right)}^{0.4}{\left(\frac{\sin\beta }{0.0896}\right)}^{1.3}$$

where $A_{s}$ is the specific catchment area and β is the degree of local slope gradient. This index was prepared based on DEM in the SAGA GIS software, and after exporting in the GIS environment it was classified into five classes: (1) 0–6.88, (2) 6.89–13.1, (3) 13.2–19.6, (4) 19.7–28.2, and (5) 28.3–87.8 (Figure 2n).

##### Land Use/Land Cover

Land use is one of the important indicators in the instability of slopes, and it affects the characteristics of the land and changes its behavior [53]. In this study, the land use layer was prepared and extracted from an Iranian land use map. Land use/cover classes in the current research are dry-farming, semi-dense forest, low-dense forest, semi-dense pasture, dense pasture, and woodland (Figure 2o).

##### Normalized Difference Vegetation Index

The normalized difference vegetation index (NDVI) factor shows the ability to detect growth and vegetation levels in an area [68,69]. It is obtained by subtracting the reflection values of red band (Red) or visible spectrum (0.6–6.7 μm) and near-infrared band (NIR) (0.7–1/1 μm). Equation (6) is used to calculate this index:

(6) $$\mathrm{NDVI}=\left(\mathrm{NIR}-\mathrm{RED}\right)/\left(\mathrm{NIR}+\mathrm{RED}\right)$$

The minimum and maximum values of this index, respectively, are (−1) and (+1). The NDVI map was produced in five classes: (1) (−0.351)–(−0.064), (2) (−0.064)–(0.008), (3) (0.008)–(0.099), (4) (0.099)–(0.260) and (5) (0.260)–(0.759) (Figure 2p).

##### Rainfall

Rainfall intensity and duration play a major role in landslide initiation [70]. Here, we obtained the rainfall data of eight meteorological stations from the Iranian Meteorological Organization. A rainfall map of the area was built with the inverse distance weighting (IDW) method with five classes: (1) 438–440, (2) 440–480, (3) 480–520, (4) and 520–560 (Figure 2q).

##### Distance to Fault

Large-scale structures such as faults and thrusts can influence the distribution of landslides [71]. In this study, distance to fault was calculated by the “Euclidean Distance” tool in ArcGIS software, in terms of distance from each pixel from the study area to the nearest fault. Based on these results, buffers were constructed around the fault with distances of 100 m, and this map was extracted into five classes: (1) 0–100, (2) 101–200, (3) 201–300, (4) 301–400, and (5) >400 (Figure 2r).

##### Distance to Roads

Both cut and fill slopes and improper road drainage structures associated with road construction can contribute to slope instability [72]. In this study, distance to road was calculated by the “Euclidean Distance” tool in ArcGIS software, in terms of distance from each pixel from the study area to the nearest road. Distance to roads was mapped with five categories: (1) 0–100, (2) 101–200, (3) 201–300, (4) 301–400, and (5) >400 m (Figure 2s).

##### Distance to Rivers

Another conditioning factor that directly impacts landslide susceptibility is distance to river. Flowing water is one of the factors increasing the potential for instability in the slopes, playing an effective role in mass movements. Distance to river was calculated by the “Euclidean Distance” tool in the ArcGIS software in meters of each pixel from the study area to the nearest stream line. The map was created with five classes: (1) 0–100, (2) 101–200, (3) 201–300, (4) 301–400, and (5) >400 m (Figure 2t).

##### Fault Density

Slope instabilities are more likely to occur in areas where the number of faults is high and particularly when the faults are active [73]. Fault density is the ratio of the total length of faults in a given watershed or a given area to the total area of the watershed or the area surrounding those faults [74]. The higher the density of faults in an area, the greater the split in rocks and the reduction in shear strength of rocks and slope constituents due to weathering. As a result, the risk of slope instability and landslides increases on the slopes [75]. Fault density was extracted with five classes: (1) 0–0.67, (2) 0.671–1.84, (3) 1.85–3.01, (4) 3.02–4.41, and (5) 4.42–7.12 km/km^2^ (Figure 2u).

##### Road Density

Road density is the ratio of the total length of roads in a given watershed or a given area to the total area of the watershed or the area surrounding those roads [76]. Although the quality of roads and drainage control are important, road density can also influence landslide occurrence [77]. Road density was calculated using the “Line density” tool in the ArcGIS software for modeling, and the factor was classified into five classes: (1) 0–0.440, (2) 0.440–1.210, (3) 1.210–1.914, (4) 1.914–2.772, and (5) 2.772–5.610 km/km^2^ (Figure 2v).

##### River Density

Another influence controlling landslides is river density [78]. River density is the ratio of the total length of rivers in a given watershed within a given area to the total area of a watershed or area containing those rivers [79]. We used the “Line density” tool in the ArcGIS software to extract five classes of river density: (1) 0–0.5551, (2) 0.5551–1.4608, (3) 1.4608–2.4542, (4) 2.4542–3.7983, (5) 3.7983–7.4505 (Figure 2w).

##### Lithology

Lithology often strongly influences slope stability [80], in part due to variable strength characteristics of certain bedrock types [81]. Therefore, to determine the susceptibility of various lithological formations to produce landslides, we extracted lithological units of the case study of Kamyaran geology sheet with a scale of 1:100,000. The number of lithological units in the study area was divided into 10 classes (Figure 2x).

##### Soil Texture

Landslides that involve soils are influenced by the type of soil they occur in [82]. Soil texture influences properties such as permeability and cohesion, which can influence the style of movement [83]. Primarily, landslides change soil features by exposing parent material (the C horizon) by removing organic mats and the horizon A [84]. Changes in soil texture occur when a landslide moves or removes various materials to a specific location [85]. From the study area, 20 soil samples in different lithological units were collected to determine soil texture using the hydrometric method. We used the soil texture triangle to classify textural groups. The soil map was created into five classes: (1) Silty Loam (2) Clay Loam (3) Loam (4) Sandy Loam (5) Silty Clay (Figure 2y).

## 3. Modeling Process

**Figure 3:**
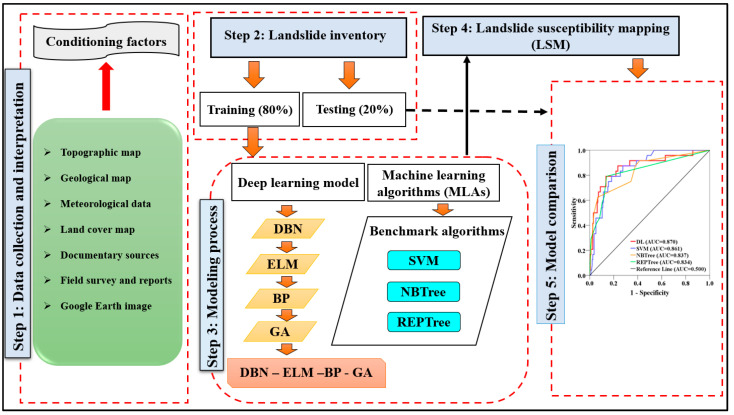
Flowchart of the study.

Figure 3 shows the workflow of our study. In step 1, we collect and interpret landslide-conditioning factors. In step 2, we divide landslide locations into the training and the validating datasets. In step 3, we conduct landslide modeling using the DL (deep learning) model and the three benchmark models (SVM, NBTree, and REPTree). In the DL model, we computed landslide susceptibility index (LSIs) for each pixel of the study in five steps: (i) constructing DBN using RBMs as pretraining on the dataset; (ii) parameter tuning in ELM to obtain the weights matrix from the last restricted Boltzmann machines (RBMs), (iii) fine tuning the training of the whole network by BP, (iv) optimizing the obtained weights from the network by the genetic algorithm (GA), and (v) assigning the optimum weights to the pixel of the study to map the landslide susceptibility. In step 4, we generate the landslide susceptibility maps using the outcomes of step 3. Finally, we compare and validate the performance of the models using a suite of statistical measures.

## 4. Mathematical Background of the Methods

### 4.1. Deep Belief Network

**Figure 4:**
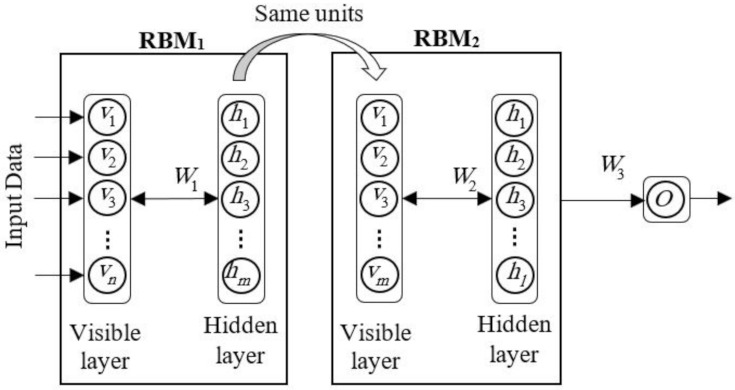
Deep belief network model used in the study.

One of the most common deep neural networks (DBN) training techniques is the use of unsupervised pretraining, which initializes the network using only unlabeled data. Network initialization has been shown to be a good starting point for fine-tuning with the next observer, and greatly reduces the risk of being trapped at the local minimum according to Kustikova and Druzhkov [86]. One of the methods used to teach deep networking is the deep belief network. The deep belief network [87,88] has become a popular approach in machine learning due to its advantages such as fast inference and the ability to encode richer and higher-order network structures. DBN operates a hierarchical structure with several finite Boltzmann machines, and operates through a layered learning process [89]. A deep belief network with two Boltzmann machines bounded for one problem to n inputs and one output is shown in (Figure 4).

Usually, with pretraining, the deep belief network training process includes the following steps [90]:

Step 1: Pretraining step: a sequential training of learning modules, greedily, one layer at a time, using unsupervised data;

Step 2: First fine-tuning step: use random weights for the last layer (matrix W3 in Figure 4);

Step 3: Second fine-tuning step: use back propagation to fine-tune the entire network using supervised data.

### 4.2. Extreme Learning Machine

The extreme learning machine (ELM) [91] was first proposed by Huang in 2004 for the single hidden-layer feedforward neural networks (SLFNs) with the aim of reducing the costs imposed by the post-error propagation procedure during the training process, and then extending to SLFNs where latent layer neurons do not need to be the same. Over the past decade, the extreme learning machine has been extensively studied due to its high productivity, effectiveness, and easy implementation [92]. The ELM has the advantage of a fast learning rate and high generalizability [93]. In ELM, the hidden layer does not need to be adjusted; that is, the connection weights from the input layer to the hidden layer as well as the hidden biases, and neurons are generated randomly without additional adjustment. The efficient least squares method is used to computationally calculate the connection weights from the hidden layer to the output layer [94].

### 4.3. Structure of the Deep-Learning Model

In the proposed model, for network training, the deep belief network training process mentioned in the DBN section is used; the difference is that in the first fine-tuning step, the ELM is applied to teach the weights between the last hidden layer and the output layer (W3 in Figure 4). The optimal network structure is also derived from GA. The steps of the genetic algorithm are as follows:

Step 1: Chromosome coding and population initialization. The chromosome is directly counted by taking positive integers (to a predetermined population N). The number of genes on each chromosome indicates the number of hidden deep layer layers and the amount of each gene indicates the number of neurons. Chromosome genes are also randomly initialized.

Step 2: Assessment. Each chromosome is trained by the proposed hybrid model using training data. Then, the classification accuracy is calculated and considered as the fit value of that chromosome.

Step 3: Selection. The known mechanism of selecting the roulette wheel has been used to choose the parents for the combination and jump.

Step 4: Combination. To search the problem space, the one-point compound operator, which is one of the most common compound operators in the literature, has been used.

Step 5: Mutation. The mutation operator produces a new chromosome by randomly selecting a gene/layer and decreasing or increasing its amount. The purpose of this operator is to prevent the algorithm from being trapped in the local optimization by discovering new solution spaces.

Step 6: Selection of survivors. After arranging the chromosomes of the current population and the chromosomes resulting from the combination and mutation based on their proportional values, the superior N chromosomes are selected as the survivors.

**Figure 5:**
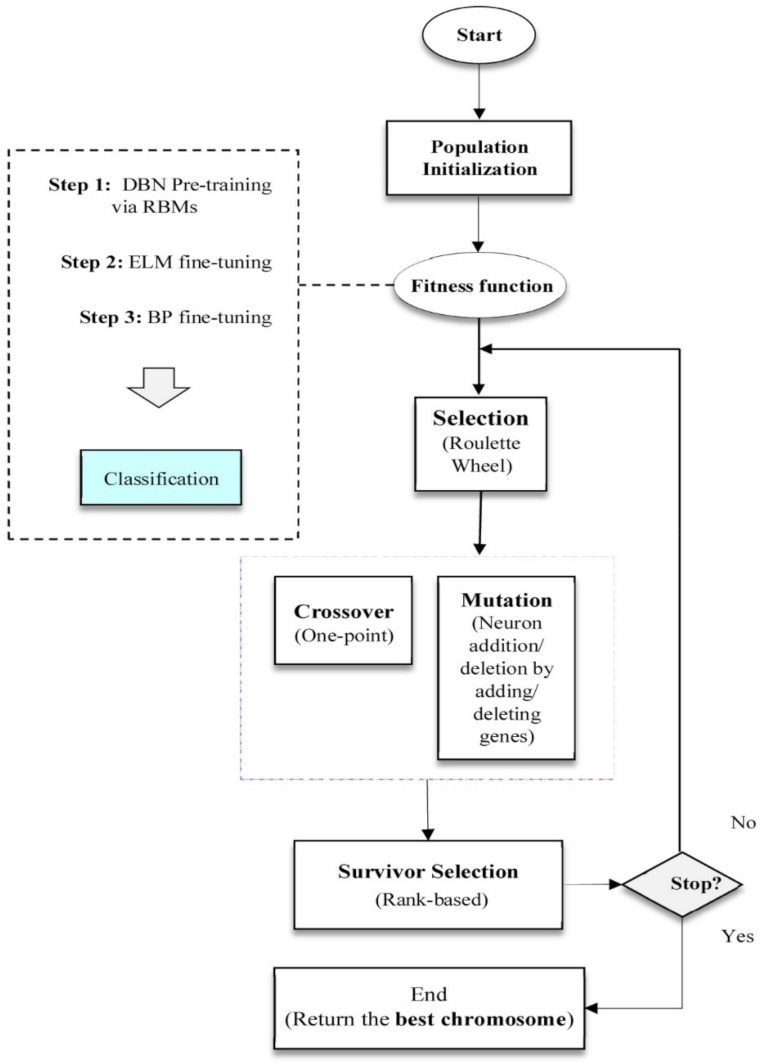
The flowchart of the deep-learning model [39,40].

Step 7: Stop criteria. When the number of generations reaches the predetermined value, the algorithm stops and the best chromosome returns as the answer; otherwise, it returns to step 3. The flowchart of the deep-learning model used in this study is shown in Figure 5.

### 4.4. Benchmark Methods

#### 4.4.1. Support Vector Machine

The support vector machine (SVM) algorithm is based on the theory of statistical learning that uses the inductive minimization principle of structural error leading to an overall optimal solution [95,96]. In recent years, this algorithm has attracted a lot of attention due to its good classification performance and good generalizability. The SVM includes the two operations, (i) nonlinear mapping of an input vector into a high-dimensional feature space that is hidden from both the input and the output and (ii) construction of an optimal hyperplane to separate the features. The structure of this model is explained as follows:

(7) $$X_{i}=\left(i=1,\;2,\;\ldots ,\;n\right)$$

The training vectors included two classes of *Y_i_* = ±1; the purpose of this model is to find a differentiated hyperplane of −*N* dimensional by the maximum gap. The description is as follows:

(8) 12=‖W‖2

Subject to the following constraints.

(9) $$Y_{i}=\left(\left(W\;.\;\;X_{i}\right)+b\right)\geq 1$$

where *‖W‖* is the norm of the normal of the hyperplane, (.) is a specific numerical production and *b* is a scalar base.

#### 4.4.2. REPTree

The reduced error-pruning tree (REPTree) as a fast decision-tree learning process that combines two kinds of algorithms as a hybrid method involving reduced-error pruning (REP) and decision tree (DT) [97]. The main structure of this method is based on classification and regression problems. The REP minimizes the complexity of tree structure if the DT’s performance is high [98]. The REPTree method uses the pruning mechanism to overcome the backward overfitting problem. Additionally, this technique uses the post-pruning method to obtain the minimal version of the most-accurate tree [99].

#### 4.4.3. NBTree

Naïve Bayes tree (NBTree) was used due to its simplicity and linear runtime method, combining the J48 algorithm and the naïve Bayes algorithm [100]. This method is used for classification problems, especially to evaluate and pick the class that maximizes the subsequent class’s likelihood. Hence, NBTree can solve problems of big data that relate to the Naïve Bayes algorithm and the data fragmentation of the J48 algorithm. The important distinguishment of this model from other machine-learning methods is that it is based on a minimal training data structure that uses a classification system to evaluate important parameters [101]. To build a Naïve Bayes classifier for detection of landslide occurrence points in the area, NBTree uses information obtained from the root node to a given leaf node down the tree, and then utilizes the training cases that fall into that leaf node [102].

### 4.5. Information Gain Ratio

In the present study, the information gain ratio (IGR) was applied as the basis of judgment for factor selection and to determine important comparative factors for modeling. For landslide susceptibility assessment, selecting the most effective factors as input dataset is fundamental. IGR was proposed by Quinlan [103] to define the quantitative predictive strength of the effective parameters and to select important conditioning factors for modeling. The higher the IGR value, the higher the prediction utility of a factor for modeling [19]. This method enhances the power of prediction of landslides, discarding noise factors with lower IGR. Assuming that the training data *T* contain *n* samples, *Ci* (landslide, nonlandslide) is a classification set of sample data, and the information entropy of the factors is calculated as follows:

(10) $$Info\;\left(T\right)=-\overset{2}{\underset{I=1}{\sum }}\frac{n\left(C_{i},T\right)}{\left|T\right|}log_{2}\frac{n\left(C_{i},T\right)}{\left|T\right|}$$

Estimating the amount of information (*T*_1_, *T*_2_ and *T_m_*) from T considering causal factor *F* takes the form of the following Equation (11):

(11) $$Info\;\left(T,F\right)=-\overset{m}{\underset{I=1}{\sum }}\frac{T_{i}}{\left|T\right|}log_{2}\;Info\;\left(T\right)$$

Eventually, the *IGR* of the landslide causal factor *F* can be calculated by:

(12) $$IGR\;\left(Y,F\right)=\frac{Info\;\left(T\right)-Info\;\left(T,F\right)}{Split\;Info\;\left(T,F\right)}$$

where *Split Info* denotes the potential information produced by dividing the training data *T* into *m* subsets. The formula of *Split Info* is shown as:

(13) $$SplitInfo\;\left(T,\;F\right)=-\overset{m}{\underset{I=1}{\sum }}\frac{\left|T_{i}\right|}{\left|T\right|}log_{2}\frac{\left|T_{i}\right|}{\left|T\right|}$$

If IGR > 1, the probability of landslide incidence is higher than average; if IGR = 0, the probability of landslide is equal to average; and if IGR < 0, the probability of landslide incidence is less than average [104].

### 4.6. Performance Metrics

To evaluate the performance of all the models, we used a number of statistical index-based metrics: sensitivity (SST), specificity (SPF), accuracy (ACC), F1-measure, and receiver operative characteristic curve (AUC). All statistical metrics were computed based on true positive (TP), true negative (TN), false positive (FP), and false negative (FAN). Table 1 shows the mentioned statistical index-based metrics and their descriptions.

## 5. Results

### 5.1. The Most Important Conditioning Factors

We obtained the relative importance of the factors influencing landslide occurrence based on average merit (AM) as IGR score through the *k*-fold cross-validation technique (Table 2). Results indicated that in the lower folds (1 and 2 folds), the number of removing factors with less predictive power was higher (13 factors) than the higher folds (10-fold; 9 factors). According to Table 2, the results pointed out that from 1-fold to 10-folds cross-validation, distance to road (AM = 0.177), road density (AM = 0.118), lithology (AM = 0.079) and land use (AM = 0.055) were the first four most important factors for landsliding in the study area. These four influencing factors are followed by NDVI (AM = 0.04), elevation (AM = 0.04), soil (AM = 0.031), aspect (AM = 0.025), solar radiation (AM = 0.021), VRM (AM = 0.015), slope angle (AM = 0.014), distance to fault (AM = 0.014), TWI (AM = 0.013), LS (AM = 0.011), TRI (AM = 0.008), and rainfall (AM = 0.006). Further, the results showed that profile curvature, curvature, plan curvature, distance to river, VD, fault density, river density, TPI, and SPI, because of having AM = 0, were removed from the modeling process.

### 5.2. Performance of the Deep-Learning Model

**Figure 6:**
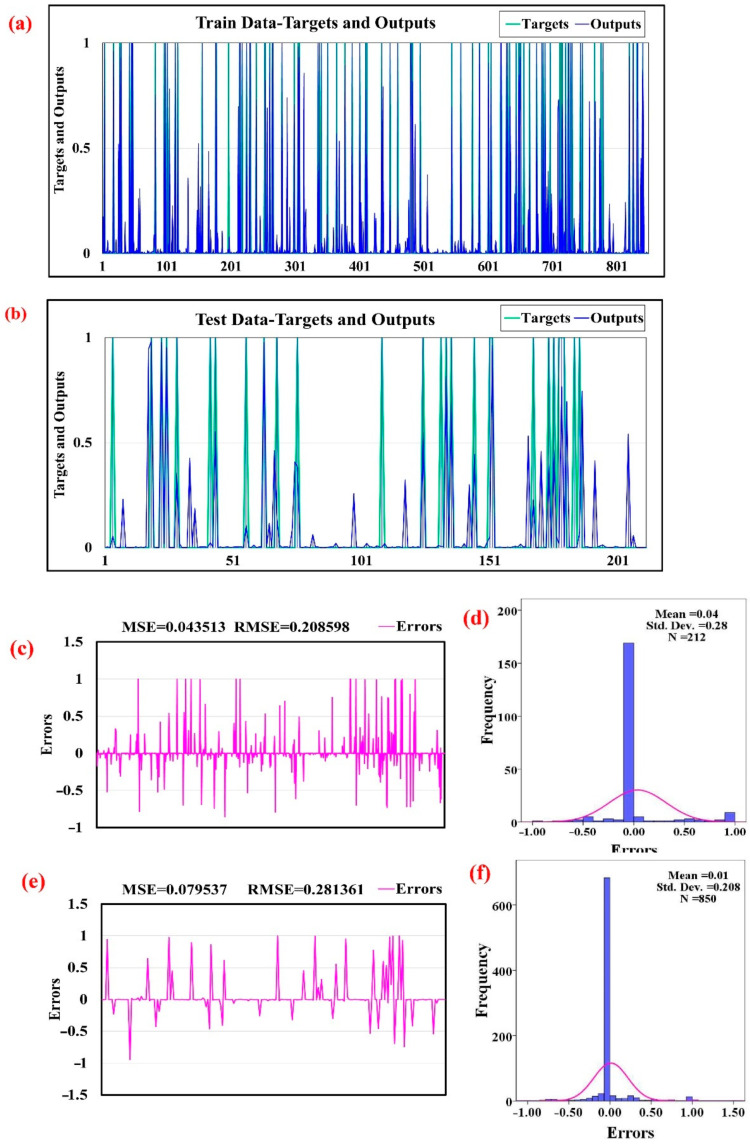
Performance of the DL model: (**a**) target and output for the training dataset, (**b**) target and output for the testing dataset, (**c**) magnitude of the errors for the training dataset, (**d**) distribution of the errors for the training dataset (**e**) magnitude of the errors for the testing dataset, (**f**) distribution of the errors for the testing dataset.

Figure 5 shows the results of training the DL model. Figure 6a,b illustrates how well the landslide (target) and nonlandslide (output) values fitted based on the training and testing datasets, respectively. A well-trained model with a high goodness of fit also has a high agreement between the target and output by the training dataset. However, high prediction accuracy of the model is inferred by the agreement between the target and output of the testing dataset. The two statistical quantitative metrics of MSE (mean squared error) and RMSE (root-mean-square error) show the modeling error of the DL model (Figure 6c,e). The values of MSE and RMSE in the training dataset were 0.0435 and 0.0208, respectively (Figure 6c); however, these values for the testing dataset were 0.079 and 0.281 (Figure 6e). The StD (standard deviation) and mean for the training dataset were, respectively, 0.04 and 0.280, and for the testing dataset these values were 0.01 and 0.208, respectively (Figure 6d,f).

#### 5.2.1. Parameter Tuning

The success rate of a model depends on selecting the optimal value of the parameters of that model. The parameter can be tuned by offline and online approaches. In the offline technique, the values of different parameters are fixed, whereas in the online approach the parameters are dynamically or adaptively controlled and updated [114]. In this study, we used the online parameter tuning approach and the results are shown in Table 3, Table 4 and Table 5.

#### 5.2.2. Classification Performance

After selecting the optimal parameter values for each model, we ran the models to obtain the highest evaluation measures but the least error. Our findings for the validation phase using the testing dataset, which are briefly reported below and in Table 6, show that the DL model outclassed and outperformed the three benchmark models. The sensitivity, specificity, accuracy, F1-measure, and AUC metrics were obtained based on the four possibilities from the confusion matrix of TP, TN, FP, and FN (Table 6).

(1) The highest sensitivity (0.667) was obtained by the DL model in which the DL model correctly classified 66.7% of landslides as landslide. It is followed by NBTree, REPTree, and SVM algorithms (Table 6).
(2) The DL model acquired the highest specificity values (0.958), indicating that the DL model correctly classified 95.83% of nonlandslides as nonlandslide. SVM, REPTree, and NBTree yielded the same specificity values based on the validation dataset of 0.953 (Table 6).
(3) The DL model had the highest accuracy on the testing dataset (0.926). It indicated that 92.6% of landslides and nonlandslides were correctly classified, respectively, as landslide and nonlandslide. It was followed by the NBTree (0.917), REPTree (0.903), and SVM (0.894) models (Table 6).
(4) The DL model had the highest value of the F1-measure (0.667), followed by NBTree (0.625), REPTree (0.533) and SVM (0.465) (Table 6).
(5) The DL model yielded the highest value of AUC (0.893) using the testing dataset. It indicated that the DL model had the highest prediction accuracy of all the models including NBTree (0.866), SVM (0.853), and REPTree (0.817) (Table 6).

### 5.3. Preparing Landslide Susceptibility Maps

**Figure 7:**
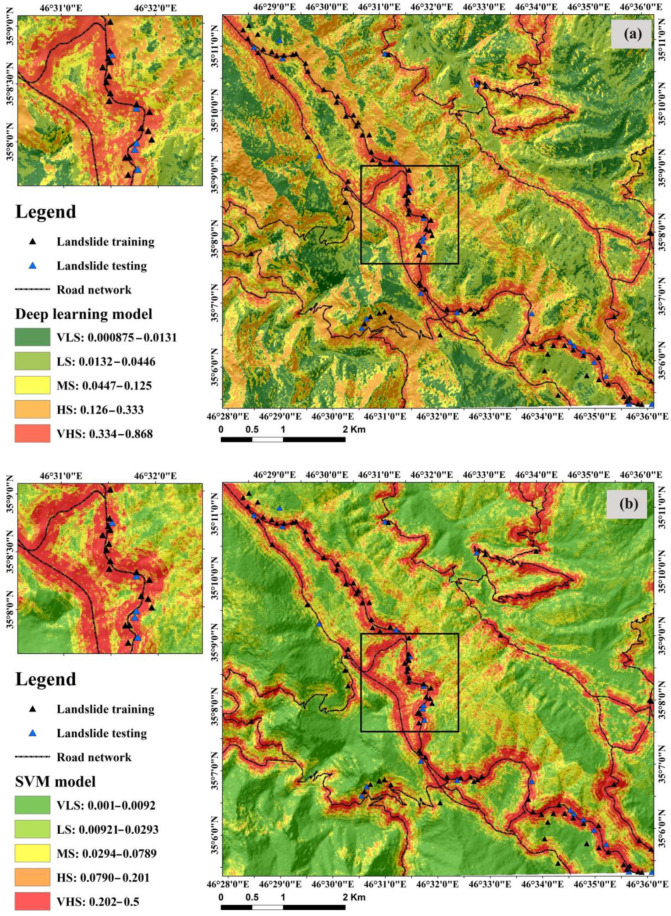

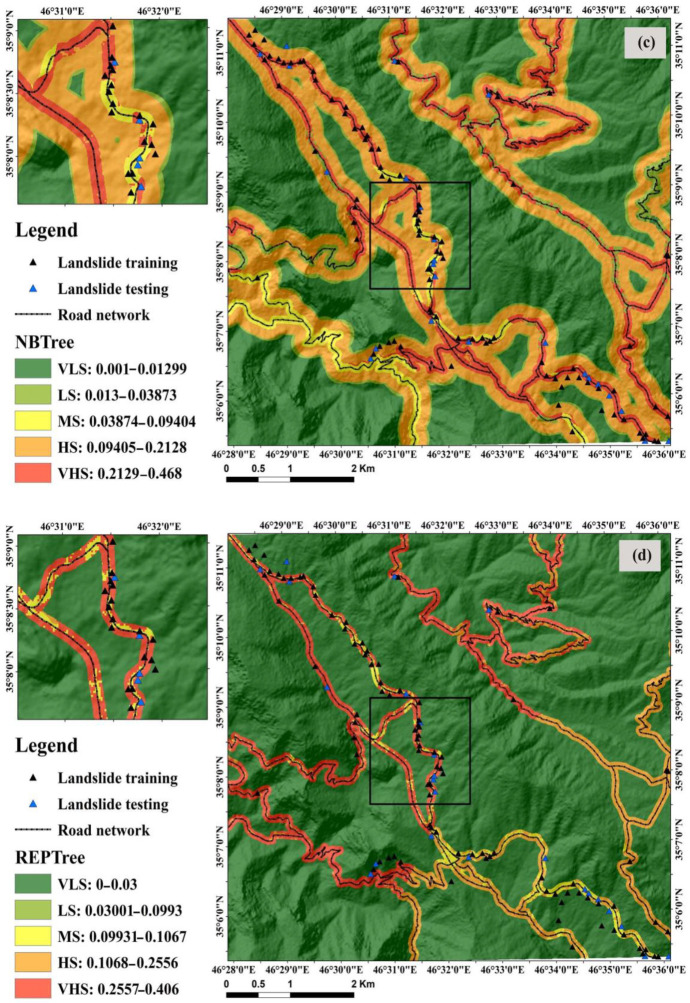
Landslide susceptibility maps produced by the (**a**) DL, (**b**) SVM, (**c**) NBTree, and (**d**) REPTree models.

We ran the DL model and also the three benchmark models (SVM, NBTree, and REPTree) and computed landslide susceptibility index (LSIs) for each pixel of the study area. We then assigned the LSIs from the DL and benchmark machine-learning models to each pixel of the study area to produce landslide susceptibility maps (Figure 7a–d). We classified the maps into the five susceptibility classes: very low susceptibility (VLS), low susceptibility (LS), moderate susceptibility (MS), high susceptibility (HS), and very high susceptibility (VHS). In DL, the range of the classes were, respectively, 0.0000875–0.0092, 0.0312–0.0446, 0.0447–0.125, 0.126–0.333, and 0.333–0.868 (Figure 7a). These classes in SVM (Figure 7b) for the VLS, LS, MS, HS, and VHS were, respectively, 0.001–0.092, 0.0921–0.0293, 0.0294–0.0789, 0.079–0.201, and 0.202–0.5. For NBTree (Figure 7c) these classes were VLS (0.001–0.01299), LS (0.013–0.03783), MS (0.03784–0.09404), HS (0.09405–0.2128), and VHS (0.02129–0.468). Consequently, in REPTree (Figure 7d) the classes were VLS (0–0.03), LS (0.03001–0.0993), MS (0.09931–0.1067), HS (0.1068–0.2556), and VHS (0.2557–0.406).

### 5.4. Validation and Comparison of the Models

**Figure 8:**
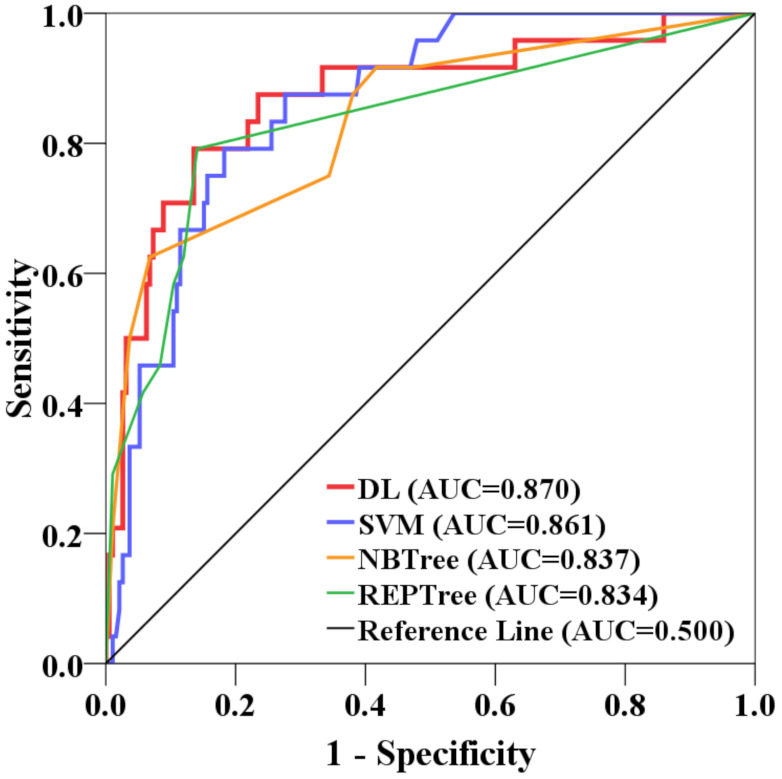
AUC of the models based on the testing dataset.

The prediction accuracy of the DL model and the benchmark machine-learning algorithms were assessed by AUC using the testing dataset (Figure 8). As shown in Figure 8, results indicated that the DL model had a high prediction accuracy (AUC = 0.870). In contrast, AUC for the SVM, NBTree, and REPTree models were somewhat lower, at, 0.861, 0.837, and 0.834, respectively (Figure 8). Overall, the DL model was superior compared to the other three benchmark machine-learning models in terms of prediction accuracy.

## 6. Discussion

In recent years, the demand for accurate prediction of landslides and the production of landslide susceptibility maps has increased, in part due to the improvement of data processing techniques, but also due to the importance of landslide prediction and susceptibility mapping in more effective land-use planning and management. There are numerous approaches and methods for producing landslide susceptibility maps, but machine-learning methods based on GIS-automated techniques offer advantages such as low cost, wide scope, fast analysis, and the option for periodically updating outputs. Each machine-learning method has its specific advantages and disadvantages, and depending on the software capabilities and input data, its outputs may differ from that of other methods. The challenge many researchers face is selecting the most appropriate method. Thus, comparative analysis of the predictive performance of different machine-learning methods is a major topic in the landslide literature [115,116]. With a desire to produce a landslide susceptibility map with high prediction accuracy, we compared the predictive performance of four machine-learning methods. We first investigated the usefulness of the conditioning factors for the modeling using the information gain ratio technique with 10-fold cross-validation. The results revealed the landslides that have occurred in our study area were significantly associated with road networks, such that the distance to roads and road density factors were identified as the most influential landslide-conditioning factors. Jaafari et al. [117] and Schlögl and Matulla [118], and Sultana and Tan [119] have also reported on the strong associations between road networks and the frequency of landslide occurrences. Therefore, these regions should be the priority targets for landslide mitigation measures [119,120].

We measured the predictive performance of the models using several widely used metrics [115,121,122] and found that the DL model has obtained the first rank in all metrics used, and therefore successfully outperformed the benchmark models (i.e., NBTree, REPTree, and SVM) that have been previously used for landslide susceptibility mapping in many regions around the world [121,122,123,124,125].

DL algorithms are powerful types of machine-learning algorithms, which utilize numerous hidden layers to model complex relationships among data for pattern recognition and classification tasks, such as landslide prediction. In contrast to traditional shallow learning algorithms (e.g., backpropagation neural networks, logistic regression, and decision trees) that generate decision boundaries directly based on the original datasets [126], DL algorithms hierarchically analyze the original datasets to extract the most relevant features for the data classification [127].

Despite the infrequent applications of the DL model for the prediction of natural hazards, the superiority of this model to other models derived from machine learning has been confirmed. For example, Wang et al. [128] reported the first application of DL for landslide prediction and achieved better prediction accuracy than that of SVM. Sameen et al. [123] reported that the DL model outperformed ANN and SVM for landslide prediction. Huang et al. [129] reported that the DL model was superior to ANN and SVM for landslide prediction. Dao et al. [130] showed that the DL model could provide a more accurate prediction of landslide susceptibility compared to quadratic discriminant analysis, Fisher’s linear discriminant analysis, and ANN. Dou et al. [131] reported that DL provided greater AUCs than the ANN and LR methods for landslide prediction. In a recent study, Mandal et al. [132] demonstrated improved accuracy for landslide prediction using the DL model compared to RF, ANN, and Bagging. Overall, the DL model has proven efficiency for landslide modeling and has been identified as an attractive alternative to traditional machine-learning methods.

## 7. Conclusions

We illustrated the robustness of a deep-learning model against three benchmark models (SVM, NBTree, and REPTree) for the prediction of landslide susceptibility in Kamyaran city, Kurdistan Province, Iran. First, the landslide inventory map with 118 past landslides was produced using different sources and randomly divided into two groups: 80% for the model training and 20% for the model validation. Next, using the models, the past landslides were linked to 25 conditioning factors. The performance of the models was evaluated using sensitivity, accuracy, specificity, F1-mesaure and AUROC. The results showed that although all models had acceptable performance, the deep-learning model outperformed the other models. This indicates that the DL model can be considered as a promising technique for preparing landslide susceptibility in mountainous areas prone to landsliding. Based on the results obtained from the deep-learning model, an accurate landslide susceptibility map is developed to complement previous research. The findings of this study can be used for future planning, land management, land use allocation, and government policy making, to prevent or reduce landslides in Kamyaran city. In future studies, we suggest integrating the current framework with other individual and metaclassifiers from machine learning to achieve a higher prediction accuracy for landslides, and perhaps other natural hazards.

## Figures and Tables

**Table 1:** Performance metrics and their descriptions to assess the performance of the models.

Metric	Formula	Description
TP	True positive	Number of landslides (positive) that are truly classified as landslide [105].
TN	True negative	Number of nonlandslides (negative) that are truly classified as nonlandslide [106].
FP	False positive	Number of nonlandslides that are incorrectly classified as landslides [107].
FN	False negative	The number of landslides that are incorrectly classified as non-landslides [9].
SST	$\mathrm{SST}=\frac{\mathrm{TP}}{\mathrm{TP}+\mathrm{FN}}$	The ratio of landslides that are correctly classified as landslide. This indicates the good predictability of the landslide model for classifying landslides [108].
SPF	$\mathrm{SPF}=\frac{\mathrm{TN}}{\mathrm{FP}+\mathrm{TN}}$	The ratio of nonlandslides that are correctly classified as non-landslide. This depicts good predictability of the landslide model for classifying nonlandslides [108].
ACC	$\mathrm{ACC}=\frac{\mathrm{TP}+\;\mathrm{TN}}{\mathrm{TP}+\mathrm{TN}+\mathrm{FN}+\mathrm{FN}}$	The ratio of landslides and nonlandslides that are correctly classified [109]. This shows how well the landslide model works.
F1-measure	$F1-\mathrm{measure}=\frac{2\mathrm{TP}}{2\mathrm{TP}+\mathrm{FP}+\mathrm{FN}}$	F-measure is a way to combine and balance both precision and recall into a single measure [110].
AUC	$\mathrm{AUROC}=\sum \mathrm{TP}+\sum \frac{\mathrm{TN}}{P}+N$	The ROC curve is plotted by sensitivity and 1-specificity, respectively, on the *y*-axis and *x*-axis [111]. The area under the ROC curve (AUC) illustrates the power prediction of a model [112].
MSE RMSE	$\mathrm{MSE}=\frac{1}{N}\overset{N}{\underset{i=1}{\sum }}{\left(x_{m}-x_{p}\right)}^{2}$ $\mathrm{RMSE}=\sqrt{\frac{1}{N}\overset{N}{\underset{i=1}{\sum }}{\left(x_{m}-x_{p}\right)}^{2}}$	MSE and RMSE measure the difference between measurements (x_m_) and predictions (x_p_) and indicate modeling error [113]

**Table 2:** Relative importance of landslide conditioning factors measured by information gain ratio technique.

Factor	1-Fold	Factors	2-Folds	Factors	3-Folds	Factors	4-Folds	Factors	5-Folds	Factors	6-Folds	Factors	7-Folds	Factors	8-Folds	Factors	9-Folds	Factors	10-Folds
DRo	0.180	DRo	0.18	DRo	0.177	DRo	0.177	DRo	0.177	DRo	0.177	DRo	0.177	DRo	0.177	DRo	0.177	DRo	0.177
RoD	0.112	RoD	0.112	RoD	0.114	RoD	0.118	RoD	0.118	RoD	0.118	RoD	0.117	RoD	0.118	RoD	0.118	RoD	0.118
Lithology	0.082	Lithology	0.084	Lithology	0.061	Lithology	0.069	Lithology	0.08	Lithology	0.079	Land use	0.055	Lithology	0.076	Lithology	0.073	Lithology	0.079
Land use	0.054	Land use	0.06	Land use	0.055	Land use	0.056	Land use	0.055	Land use	0.055	Lithology	0.07	Land use	0.055	Land use	0.055	Land use	0.055
Elevation	0.0402	Aspect	0.027	NDVI	0.041	NDVI	0.04	Elevation	0.041	NDVI	0.04	Elevation	0.04	Elevation	0.04	Elevation	0.04	NDVI	0.04
NDVI	0.0401	NDVI	0.036	Elevation	0.038	Elevation	0.04	NDVI	0.04	Elevation	0.04	NDVI	0.041	NDVI	0.04	NDVI	0.04	Elevation	0.04
Soil	0.031	Elevation	0.028	Aspect	0.024	Aspect	0.031	Soil	0.031	Soil	0.032	Soil	0.03	Soil	0.031	Soil	0.03	Soil	0.031
Aspect	0.027	SR	0.022	SR	0.021	SR	0.022	Aspect	0.024	Aspect	0.023	Aspect	0.025	Aspect	0.023	Aspect	0.026	Aspect	0.025
SR	0.020	DF	0.017	Soil	0.024	Soil	0.023	SR	0.024	SR	0.022	SR	0.022	SR	0.022	SR	0.021	SR	0.021
VRM	0.015	VRM	0.015	Slope	0.014	Slope	0.015	VRM	0.015	VRM	0.015	VRM	0.015	VRM	0.015	VRM	0.015	VRM	0.015
Slope	0. 014	TWI	0.021	VRM	0.014	VRM	0.014	Slope	0.014	Slope	0.014	DF	0.014	DF	0.014	Slope	0.014	Slope	0.014
DF	0.013	Soil	0.015	TWI	0.013	TWI	0.014	DF	0.015	DF	0.014	Slope	0.014	Slope	0.014	DF	0.014	DF	0.014
TWI	0.012	Curvature	0	DF	0.015	DF	0.012	TWI	0.013	TWI	0.013	TWI	0.013	TWI	0.013	TWI	0.013	TWI	0.013
LS	0.010	PRC	0	Curvature	0.004	Curvature	0.01	LS	0.007	LS	0.009	LS	0.01	LS	0.01	LS	0.01	LS	0.011
TRI	0.009	TRI	0	LS	0.009	LS	0	PRC	0	PRC	0	TRI	0.008	PRC	0	TRI	0.007	TRI	0.008
Rainfall	0.008	PLC	0	PLC	0.004	PLC	0	Curvature	0	Curvature	0.002	PLC	0	PLC	0	PLC	0	Rainfall	0.006
PLC	0	VD	0	TRI	0.008	TRI	0	PLC	0	PLC	0	PRC	0	Curvature	0	Curvature	0.001	PLC	0
Curvature	0	DRi	0	PRC	0	PRC	0	Rainfall	0.004	TRI	0.006	Curvature	0	TRI	0.007	Rainfall	0.006	Curvature	0
PRC	0	Slope	0	DRi	0	DRi	0	TRI	0.006	Rainfall	0.004	Rainfall	0.006	Rainfall	0.005	PRC	0	PRC	0
DRi	0	FD	0	VD	0	VD	0	DRi	0	DRi	0	DRi	0	DRi	0	DRi	0	DRi	0
VD	0	SPI	0	FD	0	FD	0	VD	0	VD	0	VD	0	VD	0	VD	0	VD	0
FD	0	TPI	0	Rainfall	0	Rainfall	0	FD	0.002	FD	0	FD	0	FD	0	FD	0	FD	0
RiD	0	Rainfall	0	TPI	0	TPI	0	TPI	0	TPI	0	TPI	0	RiD	0	RiD	0	RiD	0
TPI	0	LS	0	RiD	0	RiD	0	RiD	0	RiD	0	RiD	0	TPI	0	TPI	0	TPI	0
SPI	0	RiD	0	SPI	0	SPI	0	SPI	0	SPI	0	SPI	0	SPI	0	SPI	0	SPI	0

DRo: Distance to road; RoD: Road density; SR: Solar radiation; DF: Distance to fault; PRC: Profile curvature; PLC: Plan curvature; DRi: Distance to river; FD: Fault density; RiD: River density.

**Table 3:** The optimal value of the genetic algorithm parameters.

Parameter	Optimal Parameter Value
Number of generations	50
Population size	200
Crossover rate	0.8
Mutation rate	0.15
Number of genes	Random in (1, 5)
Value of genes	Random in (1, 200)

**Table 4:** Optimal parameters of the DBN and BP models.

Parameters	DBN	BP
	Value	Value
Learning rate	1	0.1
# of learning epochs	10	60

#: Number of...

**Table 5:** The optimal value of parameter of the benchmark methods.

Method	Parameter Value
**SVM**	Debug: False; BuildLogisticModels: False; c: 1.0; ChecksTurnedOff: False; Debug: False; Epsilon: 1.0 × 10^−12^; FilterType; Nonormalization/standardization; Kernel: Poly Kernel; NumFolds: −1; RandomSeed: 1; ToleranceParameter: 0.001
**REPTree**	Debug: False; MaxDepth: −1; MinNum: 2; MinVarianceProp: 0.001; NoPruning: False: NumFolds: 3; Seed:1
**NBTree**	Debug: False

**Table 6:** The predictive performance of the deep-learning model and the three benchmark models.

Metric	DL	SVM	REPTree	NBTree
TP	16	10	12	15
TN	184	183	183	183
FP	8	9	9	9
FN	8	14	12	9
Sensitivity	0.667	0.417	0.500	0.625
Specificity	0.958	0.953	0.953	0.953
Accuracy	0.926	0.894	0.903	0.917
F1-mesaure	0.667	0.465	0.533	0.625
AUC	0.893	0.853	0.817	0.866
